# Supplementary material for: Apigenin and Luteolin Regulate Autophagy by Targeting NRH-Quinone Oxidoreductase 2 in Liver Cells
Source: Antioxidants (Basel). 2021 May 13;10(5):776. doi: 10.3390/antiox10050776 (PMC8153271; doi:10.3390/antiox10050776)
Supplement: Supplementary file 1 [file antioxidants-10-00776-s001.zip › antioxidants-1160853-supplementary.pdf]

HepG2 experiments original blots

Apigenin and luteolin induce autophagy by targeting NRH-quinone oxidoreductase 2 in liver cells

Elzbieta Janda[1, 2*], Concetta Martino[1], Concetta Riillo[1, 2], Maddalena Parafati[1], Antonella Lascala[1], Vincenzo Mollace[1, 2], Jean A. Boutin[3]

1 Department of Health Sciences, Magna Graecia University, Campus Germaneto, Catanzaro, Italy;

2 Interregional Research Center for Food Safety and Health, Catanzaro, Italy

3 PHARMADEV (Pharmacochimie et biologie pour le développement), Université Toulouse 3 Paul Sabatier, Faculté de Pharmacie, Toulouse, France

* Correspondence: janda@unicz.it;

This supplementary document shows all original Western blots (WB) presented in Fig. 3-6 of the manuscript. The chemiluminescence signal was acquired with Chemidoc camera and converted in tiff images by Quantity One software. All blots are annotated with antigen targeted by immunoblotting and exposure time used to acquire the signal. In case of multiple signals due to sequential blots or non-specific bands, the last blotted antigen is indicated with arrows. Some blots are shown with two alternative exposure times, but more exposure times are available for all blots.

Fig. 3A (left panels)

HepG2 siNQO2 +/-Api/Lut +/-ClQ Exp02A E1304SZ

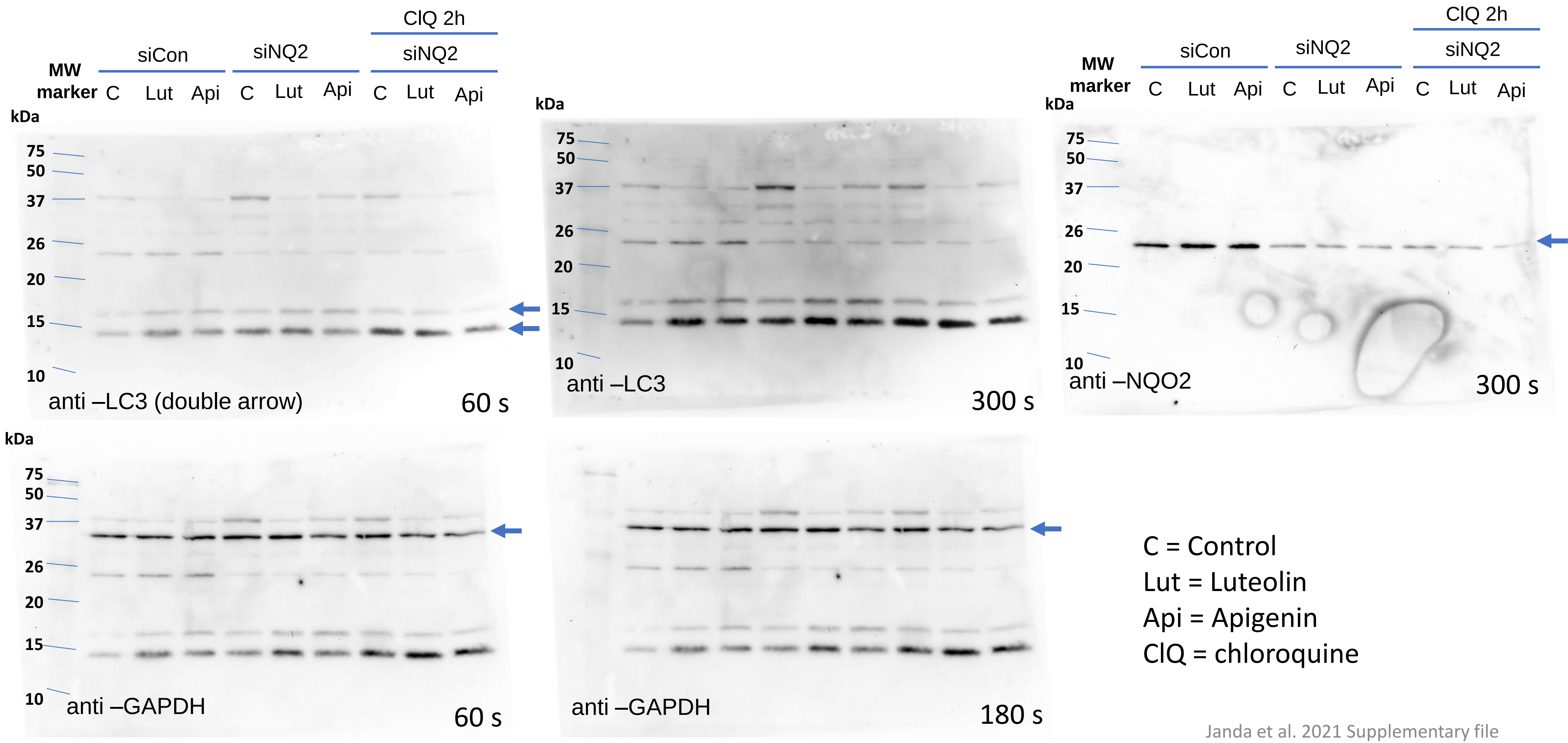

C = Control
Lut = Luteolin
Api = Apigenin
ClQ = chloroquine

Fig. 3A (right panels)

HepG2 siNQO2 +Api/Lut +ClQ Exp01B E1304SZ

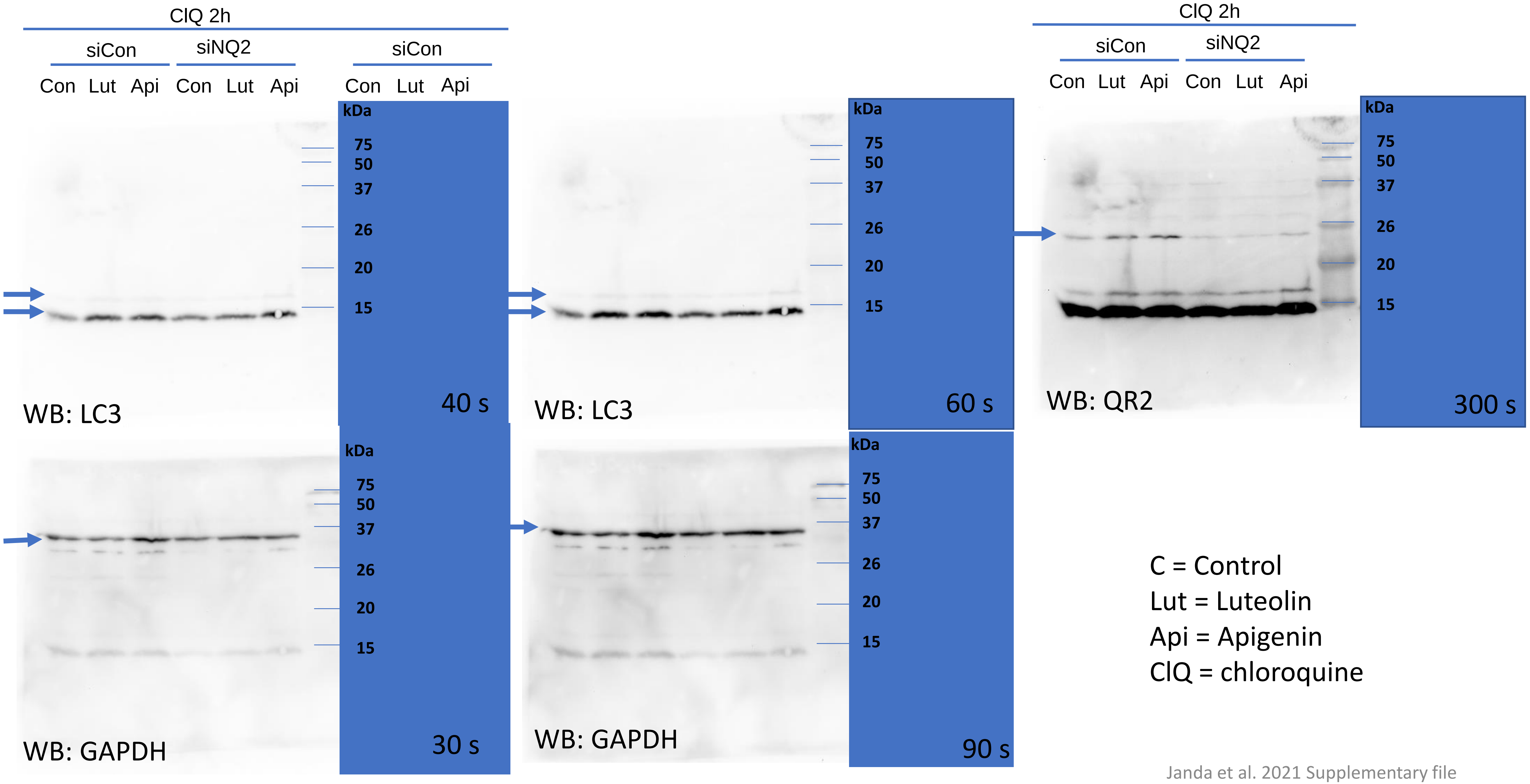

C = Control
Lut = Luteolin
Api = Apigenin
ClQ = chloroquine

HepG2 +K3 -/+ClQ_12%_1st_ GEL2

Fig. 4A
(upper panel)

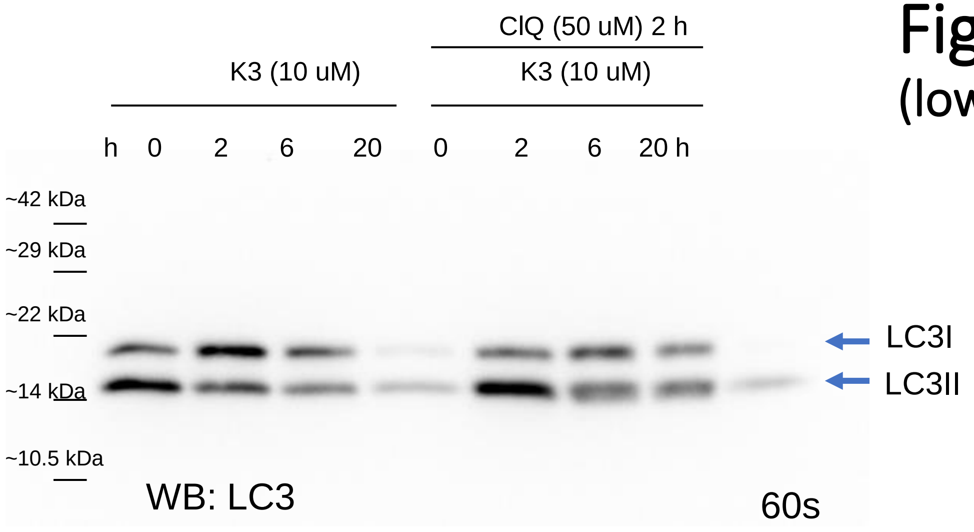

Fig. 4A
(lower panel)

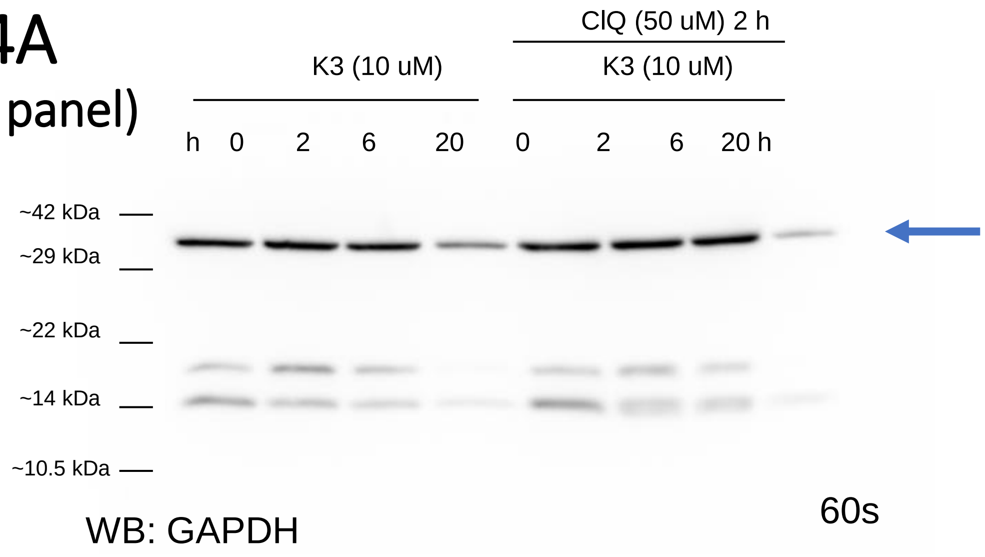

Fig. 4B
(upper panel)

HepG2 +Adrenoch -/+ClQ_12%

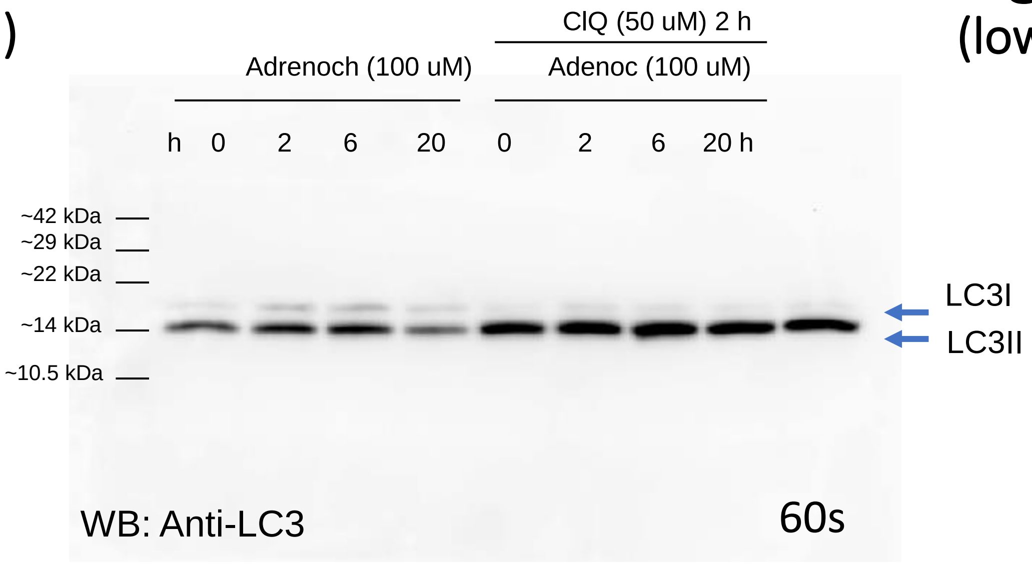

Fig. 4B
(lower panel)

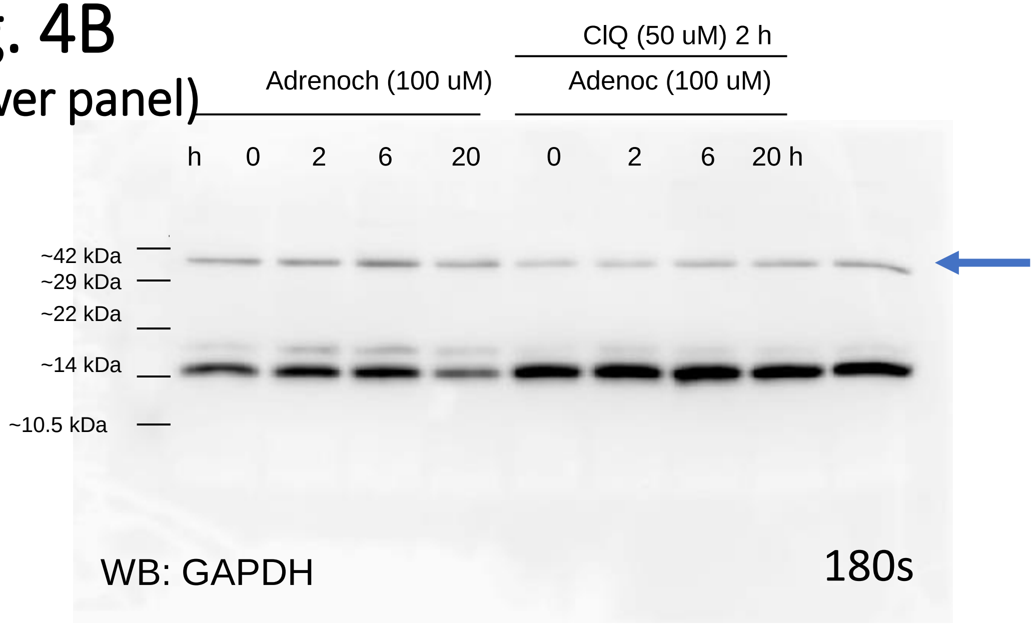

Janda et al. 2021 Supplementary file

HepG2_Adrenochrome+BNAH -/+ ClQ_12%

Fig. 4C
(upper panel)

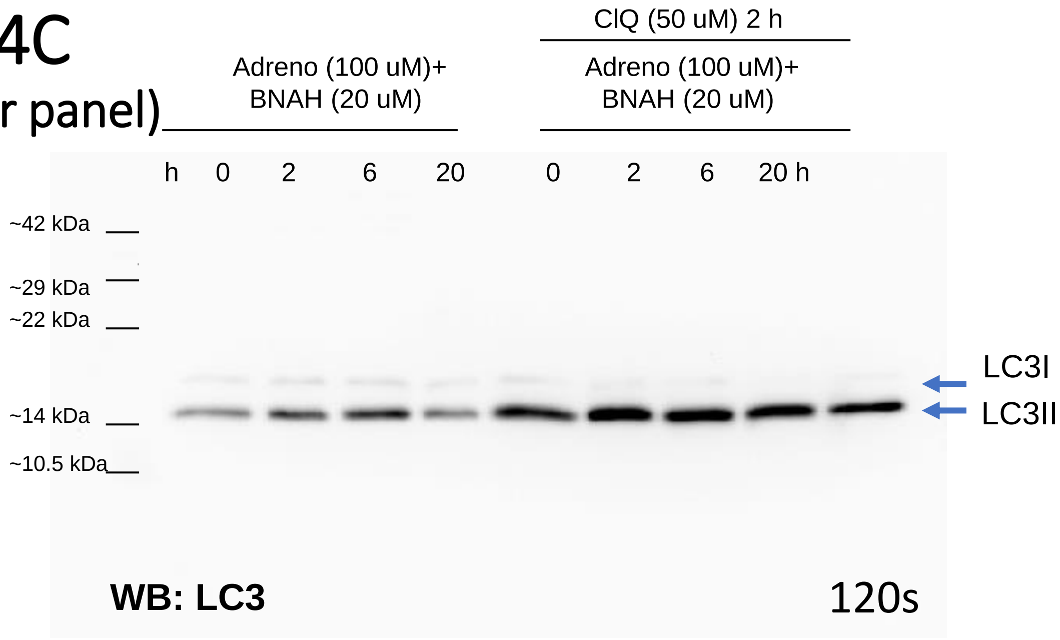

Fig. 4C
(lower panel)

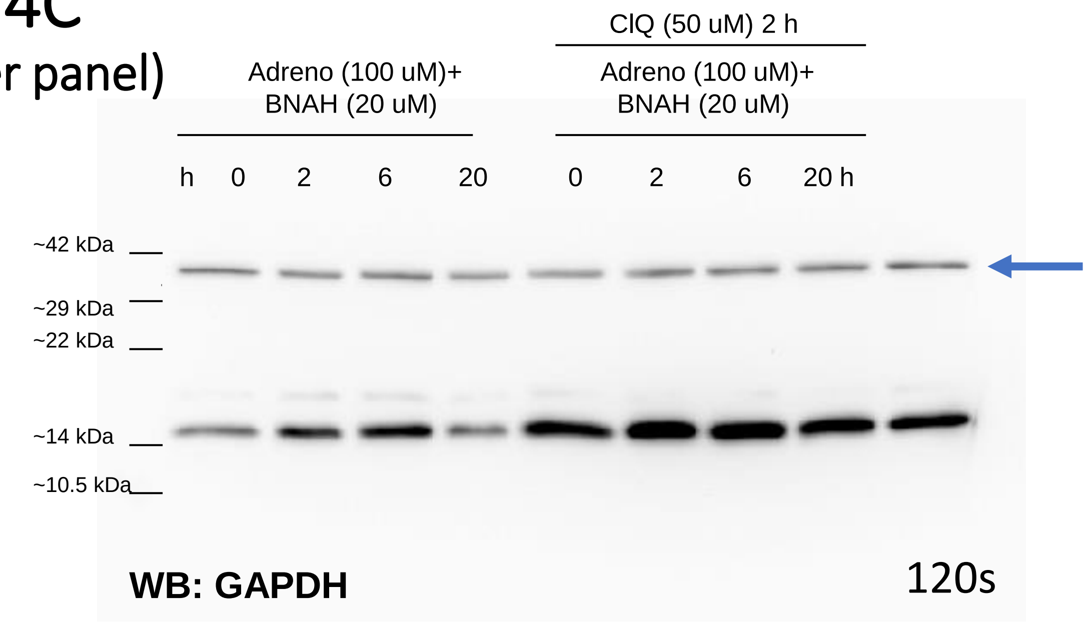

Fig. 5A

HepG2 +Lut 10/30 or +Api10/30 (6h) SDS-PAGE 10% GEL1
E2302SZ

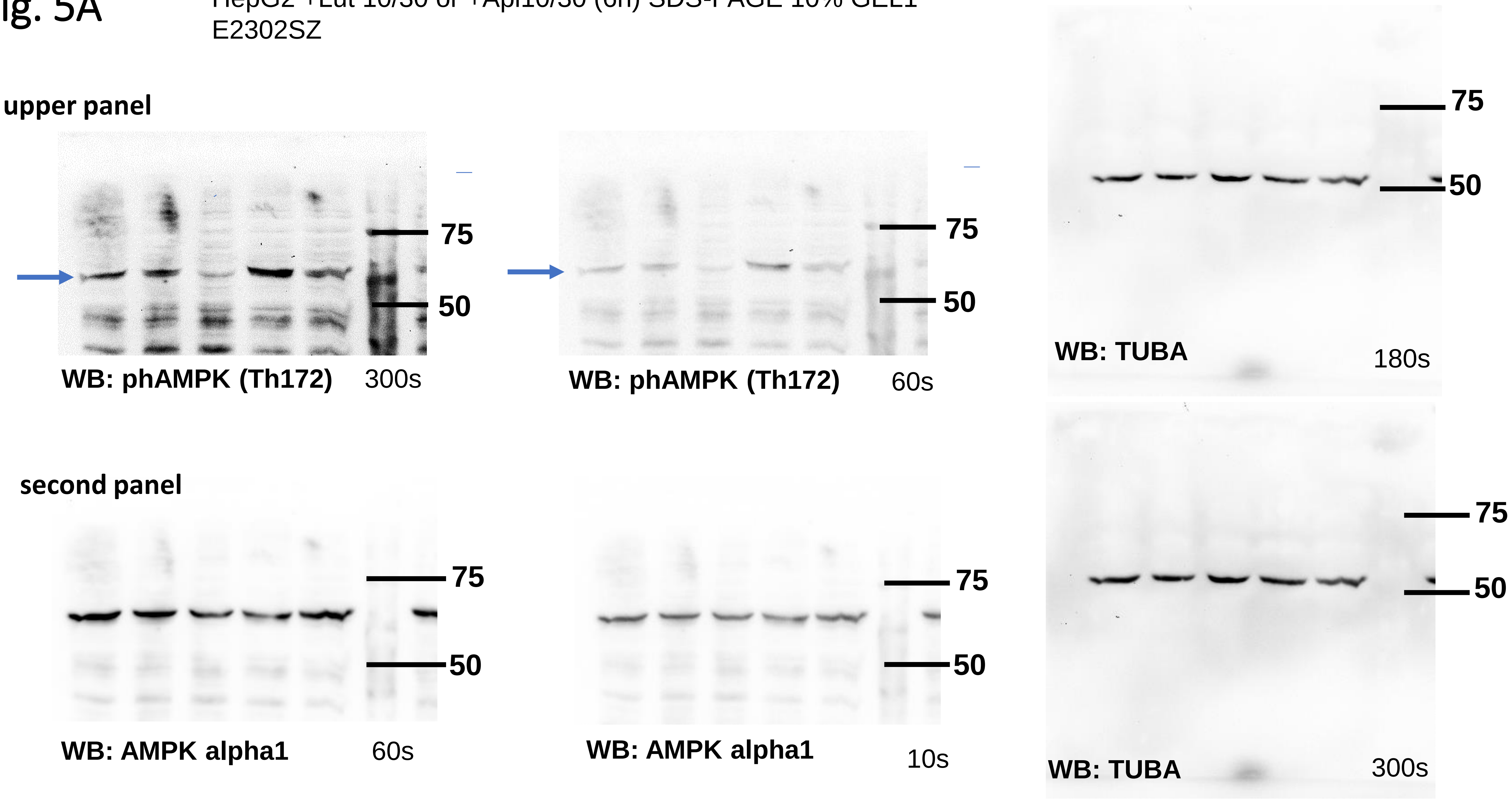

Fig. 5B

HepG2 siPRKB +/-Api/Lut E2604SZL 2704

SDS-PAGE 8%

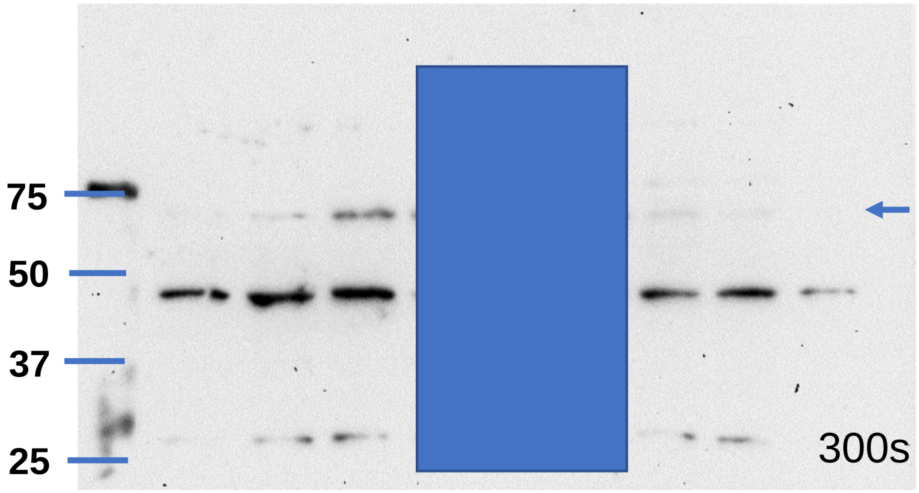

WB: anti-phAMPK (Th172) Fig. 5B, 1° upper panel

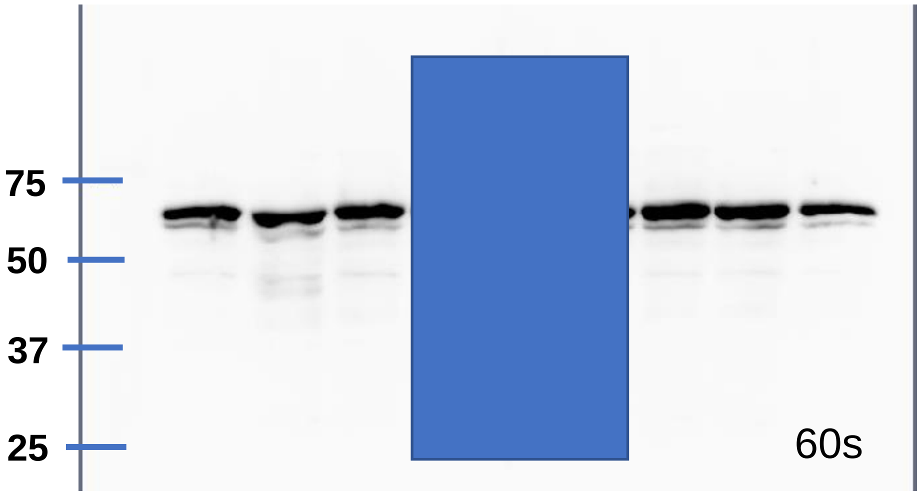

WB: anti-AMPKα Fig. 5B, 2nd upper panel

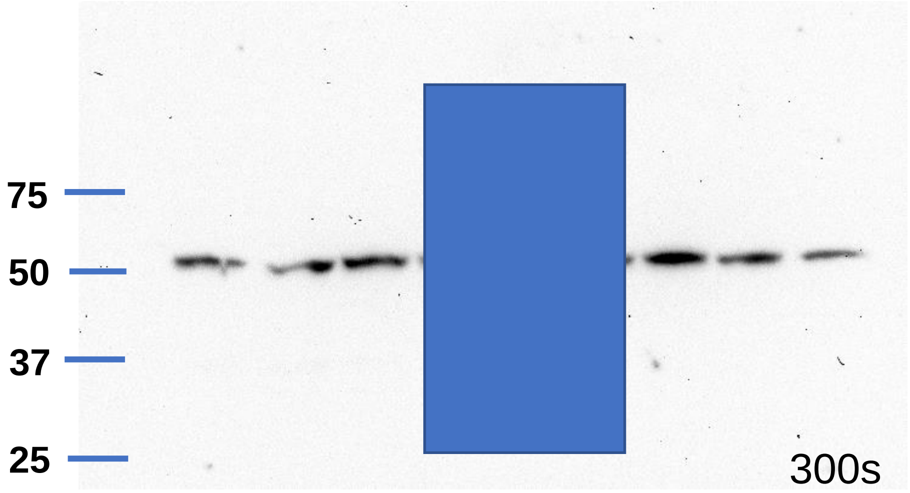

WB: TUBA Fig. 5B, 3rd panel

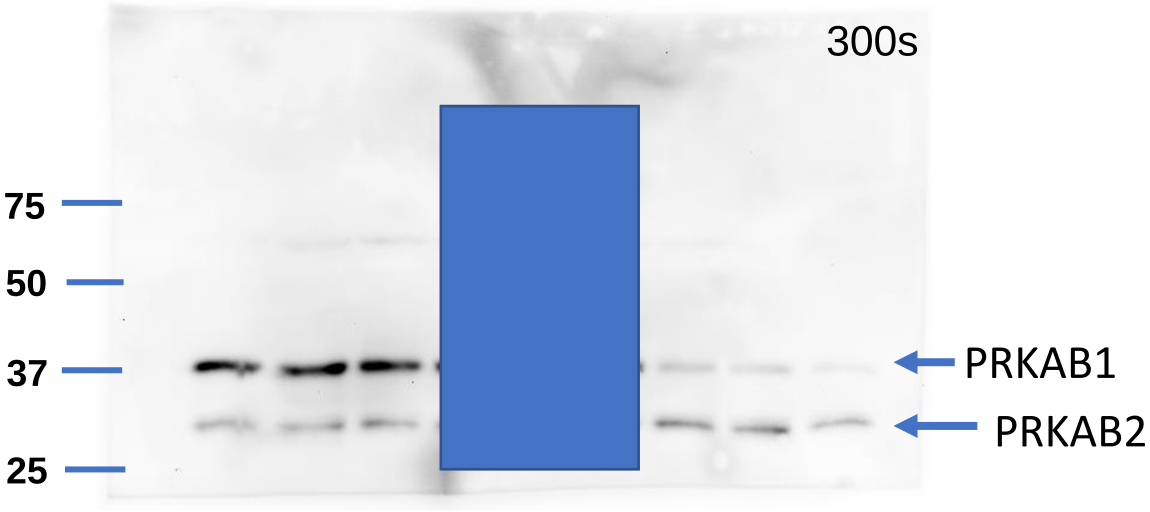

WB: anti-PRKAB Fig. 5B, 4th panel

Fig. 5B

HepG2 siPRKB +/-Api/Lut, E2604SZL 2704
SDS-PAGE 12% , GEL A

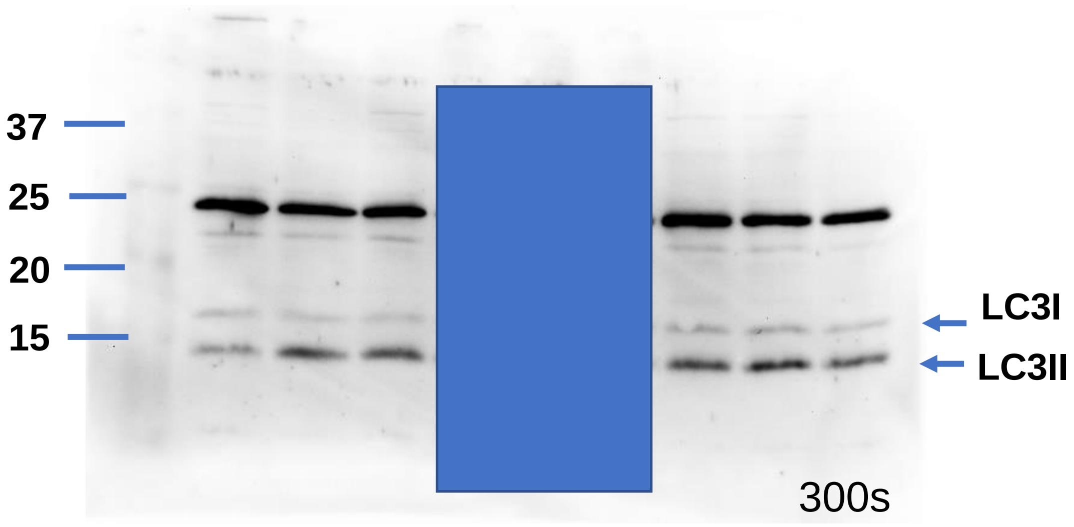

WB: anti-LC3 and anti-NQO2, Fig. 5B, 5° panel

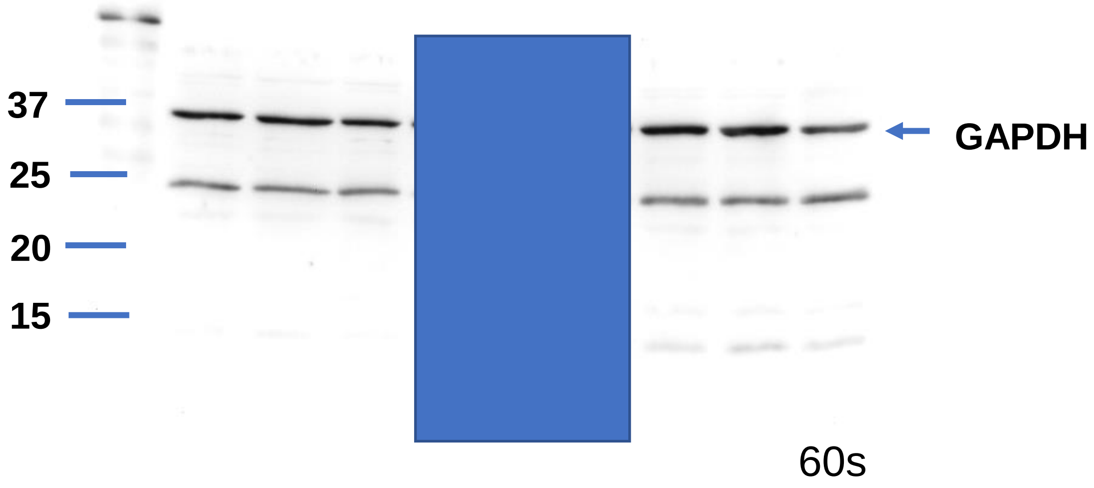

WB: anti-GAPDH (arrow) and anti-NQO2, Fig. 5B, 6th panel

HepG2 siPRKB +/-Api/Lut +ClQ E2604SZL 2704

SDS-PAGE 12% , GEL B, +ClQ

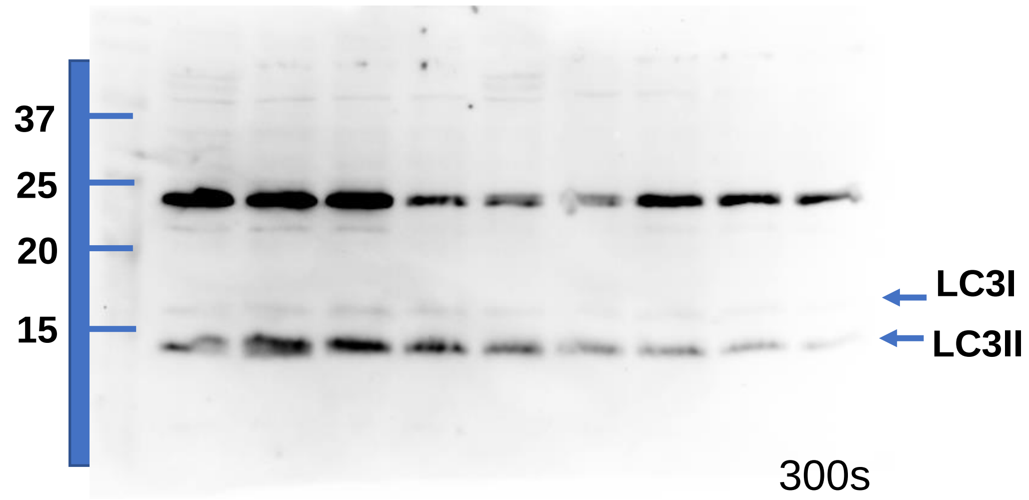

WB: anti-LC3 and anti-NQO2, Fig. 5B, 7th panel

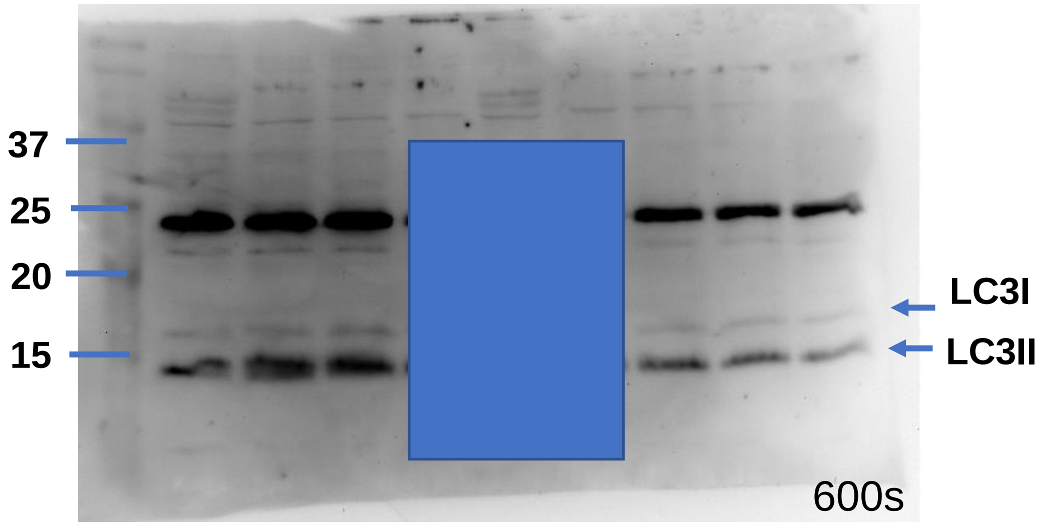

WB: anti-LC3 and anti-NQO2, Fig. 5B, 8th panel

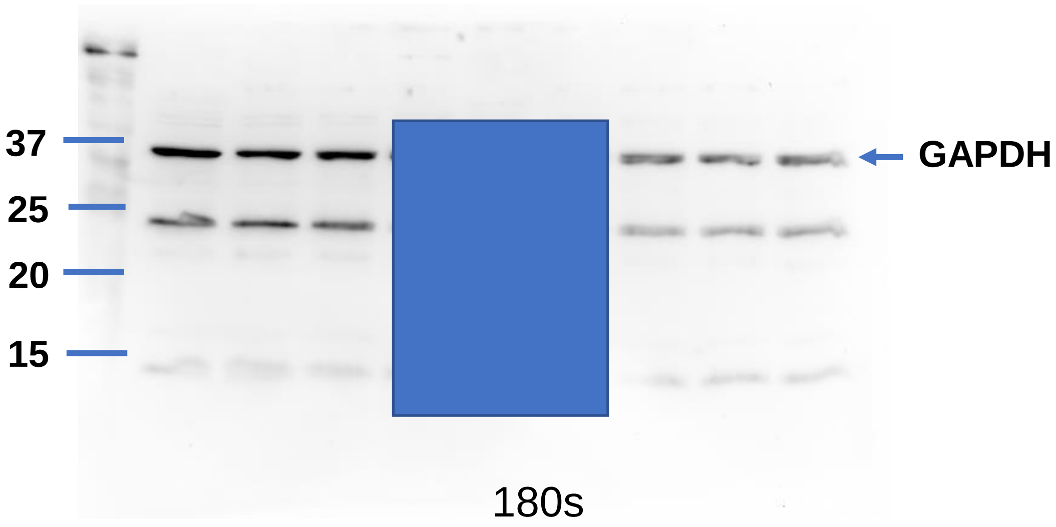

WB: anti-GAPDH (arrow) and anti-NQO2, Fig. 5B, last panel

Fig. 6

HepG2 siNQO2 +/-Api/Lut Exp03 E0104SZ

Fig. 6 SDS-PAGE 12%

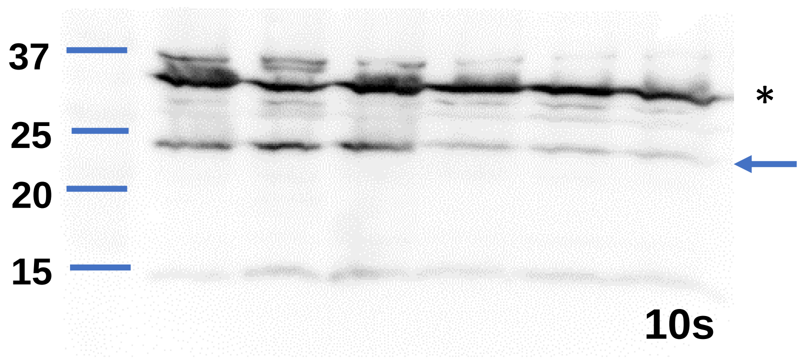

WB: anti-NQO2 (arrow) and anti-GAPDH(*), Fig. 6, upper panel 1

SDS-PAGE 8%

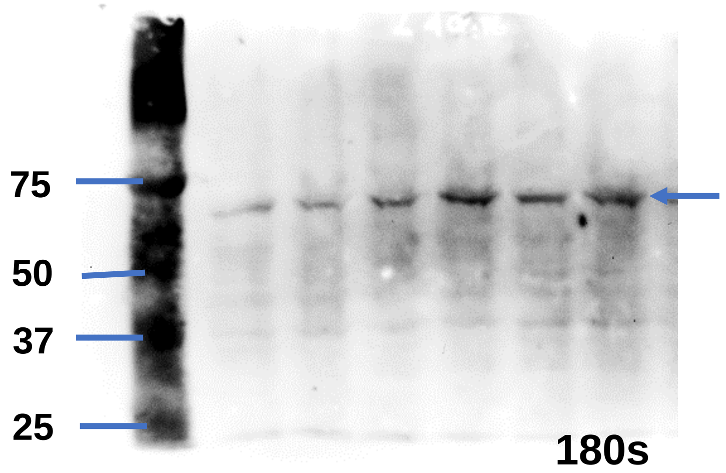

WB: anti-phAMPK Fig. 6, panel 2

SDS-PAGE 8%

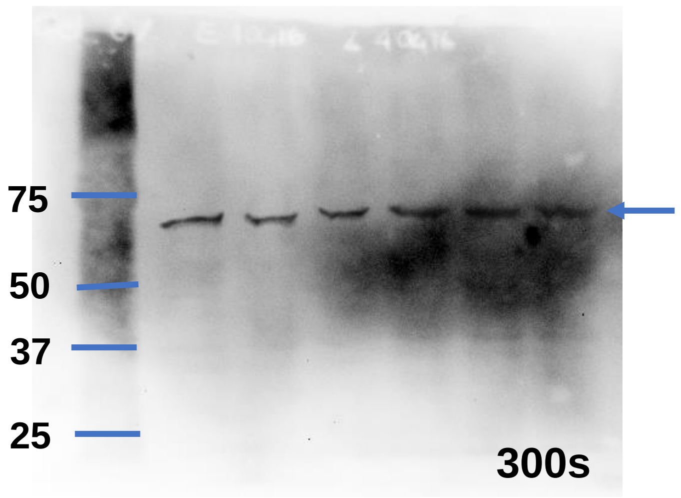

WB: anti-AMPK Fig. 6, panel 3

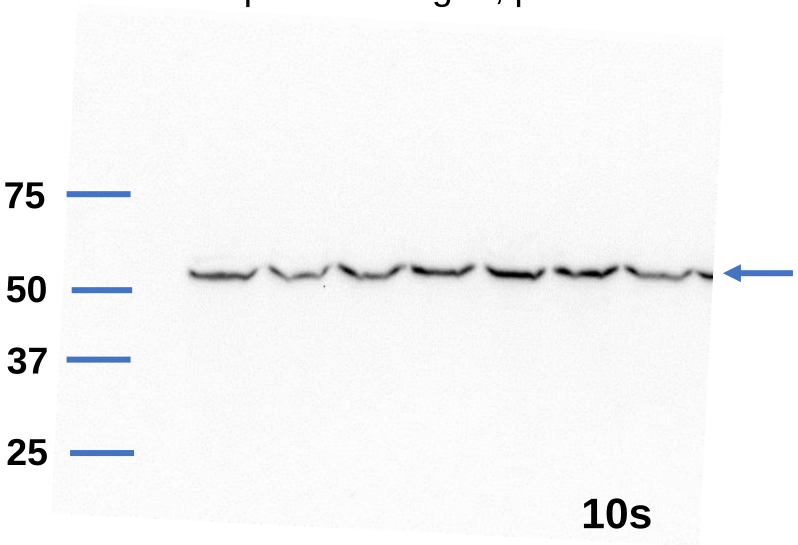

WB: anti-TUBA Fig. 6, panel 4
